# Mitigation of carbon steel biocorrosion using a green biocide enhanced by a nature-mimicking anti-biofilm peptide in a flow loop

**DOI:** 10.1186/s40643-022-00553-z

**Published:** 2022-06-13

**Authors:** Di Wang, Tuba Unsal, Sith Kumseranee, Suchada Punpruk, Mazen A. Saleh, Mohammed D. Alotaibi, Dake Xu, Tingyue Gu

**Affiliations:** 1 Shenyang National Lab for Materials Science, Northeastern University, Shenyang, 110819 China; 2 Department of Chemical & Biomolecular Engineering, Institute for Corrosion and Multiphase Technology, Ohio University, Athens, 45701 USA; 3 Institute of Marine Sciences and Management, Istanbul University, Istanbul, 34134 Turkey; 4 PTT Exploration and Production, Bangkok, 10900 Thailand; 5 Research and Development Center, Saudi Arabian Oil Company, Dhahran, 31311 Saudi Arabia

**Keywords:** Microbiologically influenced corrosion (MIC), Biofilm, Flow loop, Biocide, Biocide enhancer, Peptide

## Abstract

Biocorrosion, also called microbiologically influenced corrosion (MIC), is a common operational threat to many industrial processes. It threatens carbon steel, stainless steel and many other metals. In the bioprocessing industry, reactor vessels in biomass processing and bioleaching are prone to MIC. MIC is caused by biofilms. The formation and morphology of biofilms can be impacted by fluid flow. Fluid velocity affects biocide distribution and MIC. Thus, assessing the efficacy of a biocide for the mitigation of MIC under flow condition is desired before a field trial. In this work, a benchtop closed flow loop bioreactor design was used to investigate the biocide mitigation of MIC of C1018 carbon steel at 25 °C for 7 days using enriched artificial seawater. An oilfield biofilm consortium was analyzed using metagenomics. The biofilm consortium was grown anaerobically in the flow loop which had a holding vessel for the culture medium and a chamber to hold C1018 carbon steel coupons. Peptide A (codename) was a chemically synthesized cyclic 14-mer (cys-ser-val-pro-tyr-asp-tyr-asn-trp-tyr-ser-asn-trp-cys) with its core 12-mer sequence originated from a biofilm dispersing protein secreted by a sea anemone which possesses a biofilm-free exterior. It was used as a biocide enhancer. The combination of 50 ppm (w/w) THPS (tetrakis hydroxymethyl phosphonium sulfate) biocide + 100 nM (180 ppb by mass) Peptide A resulted in extra 1-log reduction in the sulfate reducing bacteria (SRB) sessile cell count and the acid producing bacteria (APB) sessile cell count compared to 50 ppm THPS alone treatment. Furthermore, with the enhancement of 100 nM Peptide A, extra 44% reduction in weight loss and 36% abatement in corrosion pit depth were achieved compared to 50 ppm THPS alone treatment.

**Graphical Abstract:**

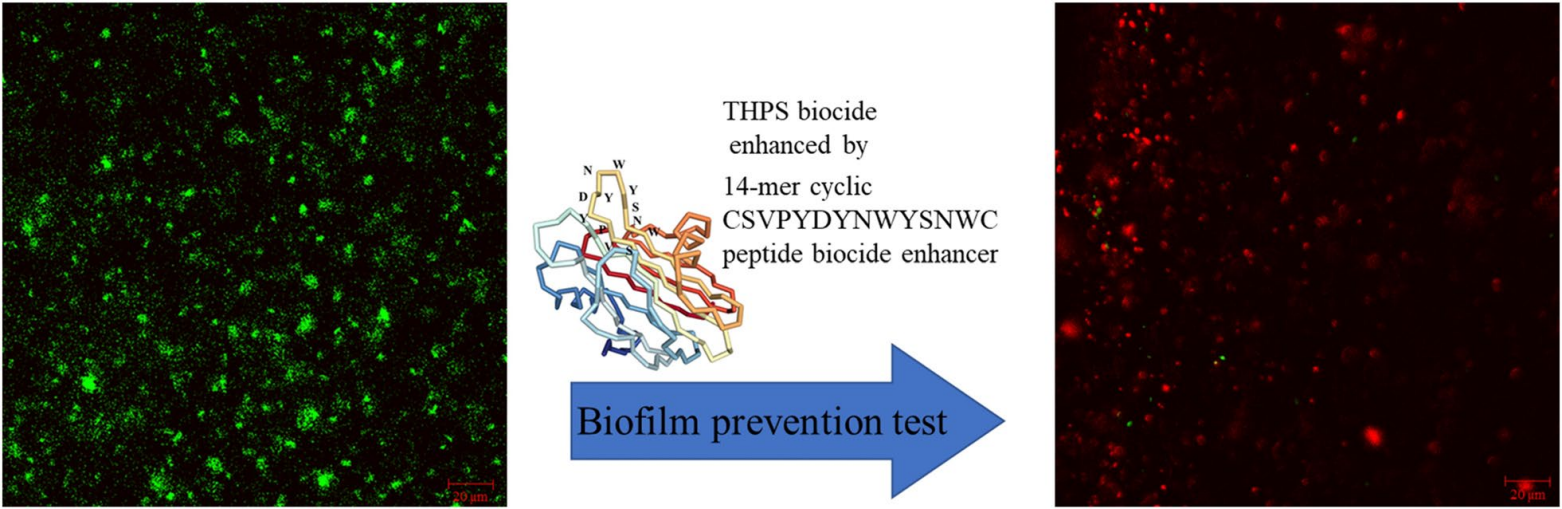

## Introduction

MIC (microbiologically influenced corrosion) is a common threat to many industrial processes and settings, such as oil and gas operations (Rasheed et al. 2019), storage tanks (Parthipan et al. 2018), reactors including those used to process biomass and those used to bioleach ores (Chang et al. 2014; Kumar and Sharma 2017; Dong et al. 2018; Kaliyaraj et al. 2019; Wu et al. 2021; Bimestre et al. 2022), biomedical implants (Kabir et al. 2021), etc. It reduces the service life of oil and gas pipelines and can potentially lead to catastrophes (Gieg et al. 2011; Qian et al. 2018; Wei et al. 2021). Microbes blamed for pipeline MIC include sulfate reducing bacteria (SRB), acid producing bacteria (APB), acetogenic bacteria and methanogens (Sherar et al. 2011; Park et al. 2011). In field operations, SRB, APB and other microorganisms usually co-exist in a biofilm consortium (Wang et al. 2020b). SRB biofilms are often the main culprit of MIC due to their prevalence in the field environments, such as oil and gas pipelines which are usually kept oxygen free. They can utilize sulfate as the terminal electron acceptor for the oxidation of an carbon source or H_2_ as the electron donor (Gu et al. 2021; Vigneron et al. 2016; Wang et al. 2020a). When there is a lack of electron donor, electroactive SRB sessile cells can utilize elemental iron as an alternate electron donor, causing corrosion (Jia et al. 2019a). Corrosive APB biofilms are also a contributing factor in an acidic environment, such as bioleaching bioreactors (Ahmadi et al. 2010; Yang et al. 2020), because they release H^+^ which is an oxidant (Kip and van Veen 2015).

Biofilm formation is a complex and continuous biological process (Donlan 2002; Tribedi et al. 2015; Tang et al. 2021). The formation and structure of a biofilm can be influenced by fluid flow velocity (Liu et al. 2017). The hydrodynamics and the mechanical properties of the biofilm can impact the interactions between the bulk fluid and the attached biofilm in a flow system (Stoodley et al. 2002). The type of fluid flow is also a factor that can impact MIC. For example, compared with laminar flow, turbulent flow can lead to more severe MIC in welded joint in a pipeline (Liduino et al. 2019). Below the threshold of 3 m/s, biofilms are believed to form on pipe walls (Song et al. 2016). *Psudoalteromonas piscicida* biofilm can induce wider and deeper cracks in horizontal flow condition than in orbital shaking condition (Moradi et al. 2019). Thus, it is necessary to test biocide mitigation of MIC in a flow system before a field trial on an actual operational process after initial biocide testing in anaerobic vials in static incubation condition.

Biocides are often used to mitigate biofilms, but large biocide dosages used in biofilm treatment suffer from high operational costs and adverse environmental impacts (Rasheed et al. 2019; Li et al. 2022). Glutaraldehyde together with tetrakis hydroxymethyl phosphonium sulfate (THPS) are the two most popular green biocides in the oil and gas industry owing to their broad-spectrum efficacy and readily biodegradable advantage (Sharma et al. 2018; Wang et al. 2020b, 2022a; Unsal et al. 2021; Kijkla et al. 2021). THPS is a broad-spectrum biocide that works by damaging cell membrane, cleaving S–S bonds in the disulfide amino acids of the cell wall (Kahrilas et al. 2015; Sharma et al. 2018). However, prolonged uses induce microbial resistance as more resistant microbes in the environment move in to fill the void left behind by vulnerable microbes.

Sessile cells embedded in a biofilm are harder to eradicate than their planktonic counterparts because biofilms can deploy several defense strategies that include diffusional limitation, and lowered metabolic rate to minimize intakes (Li et al. 2016). Some eco-friendly chemicals such as catechin hydrate and zinc pyrithione have effective inhibition on MIC, but the industrial applications of these anti-bacterial inhibitors are still not in existence (Wang et al. 2019; Lekbach et al. 2019). Thus, a more effective and economic biocide treatment strategy is desired to treat MIC.

Using a biocide enhancer is a potential strategy in the mitigation of biofilms, because they can work synergistically with existing biocides (Kahrilas et al. 2015; Li et al. 2016). Kolodkin-Gal et al. (2010) demonstrated that several d-amino acids dispersed *Bacillus subtilis* biofilm by impacting the function of amyloid fibers. In various lab experiments, d-amino acids were shown to enhance THPS and a few other non-oxidizing biocides against mixed-culture biofilms from oilfield and cooling water towers (Li et al. 2016). Biocide enhancers can be used to improve the efficacy of a biocide in various ways and they do not have to be biocidal themselves (Wang et al. 2020b; Unsal et al. 2021). Using antimicrobial agents including antimicrobial peptides and silver nanoparticles has potential drawbacks such as a biofilm becoming possibly resistant to the agents later on because their antimicrobial actions kill the weak microbes and allow resistant microbes from the surroundings to take their place (Barapatre et al. 2016; Kumar and Sharma 2017; Di Somma et al. 2020; Hussein et al. 2020; Tan et al. 2019). Non-biocidal Peptide A is an attractive biocide enhancer candidate. It has a cyclic 14-mer sequence (cys-ser-val-pro-tyr-asp-tyr-asn-trp-tyr-ser-asn-trp-cys) with its core 12-mer sequence derived from Equinatoxin II protein, which is secreted by a sea anemone to maintain a biofilm-free exterior (Zlotkin 2016). Peptide A at a very low dosage can not only inhibit the settlement of biofilms, but also disperse pre-established biofilms in the presence of a biocidal stress as demonstrated in static vial tests (Jia et al. 2019b). The addition of 180 ppb Peptide A to 2,2-dibromo-3-nitrilpropioamide (DBNPA) showed enhanced sessile reduction and MIC abatement in 60-day incubation compared to DBNPA alone treatment in static vial tests (Wang et al. 2020b).

This work tested Peptide A at a very small dosage (100 nM, 180 ppb by mass) to enhance THPS in the mitigation of a corrosive oilfield biofilm consortium in a closed flow loop bioreactor. This work aimed to show the efficacy of THPS and the enhancement by Peptide A on THPS in the prevention of biofilm establishment as well as abatement of C1018 carbon steel MIC by an oilfield biofilm consortium in flow condition. C1018 carbon steel coupons were immersed in enriched artificial seawater (EASW) inoculated with the biofilm consortium at 25 °C for 7 days under anaerobic condition.

## Materials and methods

A recalcitrant and corrosive oilfield biofilm labelled as Consortium IIe was used in this work. Its metagenomic data are listed in Table 1. An EASW culture medium was used to culture Consortium IIe biofilm. The EASW composition was (g/L): NaCl 23.476, Na_2_SO_4_ 3.917, NaHCO_3_ 0.192, KBr 0.096, KCl 0.664, H_3_BO_3_ 0.026, SrCl_2_·6H_2_O 0.040, CaCl_2_·2H_2_O 1.469, MgCl_2_·6H_2_O 10.610, yeast extract 1, tri-sodium citrate 0.5, sodium lactate 3.5, CaSO_4_·0.5H_2_O 0.1, NH_4_Cl 0.1, MgSO_4_·7H_2_O 0.71, K_2_HPO_4_ 0.05, Fe(NH_4_)_2_(SO_4_)_2_·6H_2_O 1.38. The purity was ≥ 99% for all the chemicals used in EASW. The initial pH of EASW was neutralized to 7.0 by adding a 5% (*w/w*) NaOH. EASW and lab tools such as tweezers were autoclave sterilized. EASW was then sparged with filtered N_2_ to deoxygenate with the help of a gas distributor and magnetic stirring bar for at least 1 h. One hundred ppm (*w*/*w*) l-cysteine was added as an oxygen scavenger to reduce dissolved O_2_ further. Peptide A (100% purity), chemically synthesized by Bachem AG (Bubendorf, Switzerland) and 75% (*v*/*v*) THPS (Sigma-Aldrich, St. Louis, MO, USA) were dissolved in deionized water to make a stock solution that was filtered-sterilized.

**Table 1:** Metagenomics of dominant microbial species in Consortium IIe

Microbe	%
*Garciella* spp.	92.1
*Desulfovibrio vulgaris*	3.1
*Bacillales* spp.	3.0
*Tissierella* spp.	0.88
*Thermoanaerobacterales* spp.	0.13
*Porphyromonadaceae* spp.	0.11
*Sphingomonas* spp.	0.07
Unknown	0.63

C1018 carbon steel coupons with a 1.2 cm^2^ exposed top face were tested. The metal’s mass composition was (%): C 0.20, P 0.04, Mn 1.40, Cu 0.55, S 0.04, Ni 0.012, and Fe balance. The unexposed coupon faces were painted with inert Teflon. The coupons were abraded to a 600 grit finish, and then degreased with pure isopropanol and subsequently dried under UV light. The test matrix is shown in Table 2.

**Table 2:** Test matrix of biofilm prevention test in a flow loop bioreactor

Microorganism	Consortium IIe
Cultural medium	EASW
Metal	C1018 carbon steel
Treatment methods	No treatment control
	50 ppm THPS
	50 ppm THPS + 100 nM Peptide A
	100 ppm THPS
Temperature	25 °C
Flow rate	1.2 L/h
Incubation duration	7 day
Analysis	Sessile cell count, weight loss, CLSM biofilm image, SEM corrosion pit image, pit depth

**Fig. 1:**
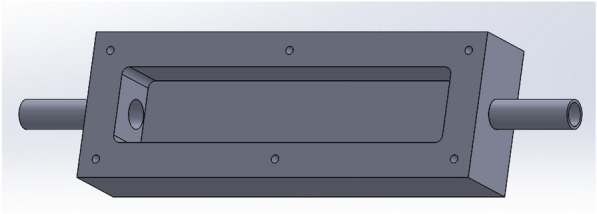
Schematic of a custom-made flow chamber to hold coupons (inner dimensions: 12.0 cm *H* × 2.5 cm *W* × 1.8 cm *D*)

**Fig. 2:**
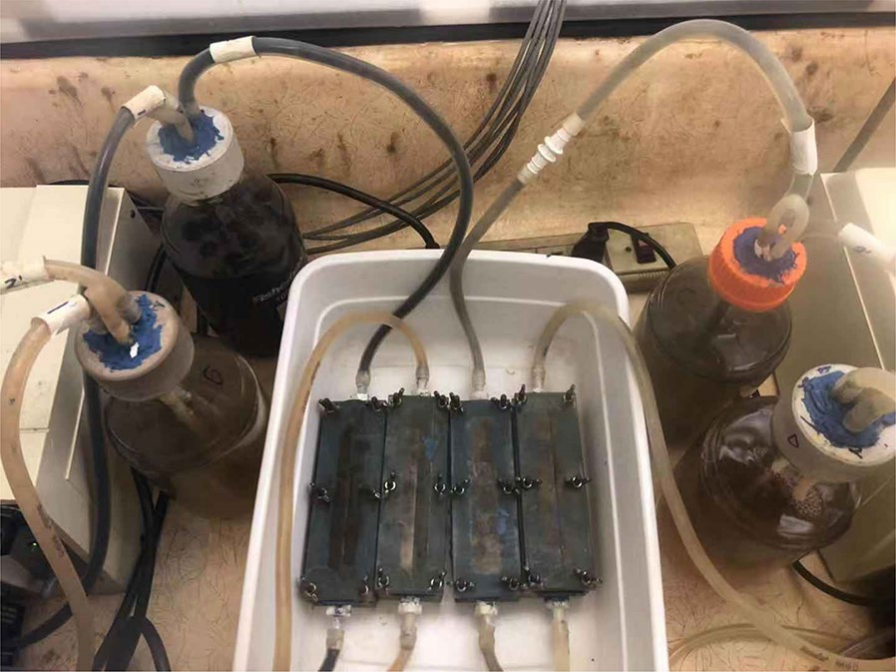
Biofilm prevention test using different biocide treatments in four independently run flow loop bioreactors

A self-fabricated acrylic flow chamber (Fig. 1) was used in this work to simulate flow condition in a re-circulating bioreactor. The carved-out inner rectangular chamber had dimensions of 12.0 cm H × 2.5 cm W × 1.8 cm D. Ten coupons were placed in the flow chamber and the chamber was sealed with a lid with a rubber gasket fastened by six screws. Silicone glue covered the seams and tubing joints to prevent any leaks. A 10 mL Consortium IIe seed culture and biocide treatment chemicals were added to 1 L EASW medium in the 1 L anaerobic bottle that was used as the holding tank in each flow loop. Then, flow was started using a peristaltic pump (Model 7520–60, Barnant Co., Barrington, IL, USA). The re-circulation flow rate was 1.2 L/h. The entire bioreactor was placed in an anaerobic chamber (Model 818-GB, PLAS Labs, Lansing, MI, USA). This chamber was sparged with sterile N_2_ for 0.75 h to achieve anaerobic condition before use. Figure 2 shows the setup for 4 independent flow loop bioreactors with a multi-channel peristaltic pump and four 1-L bottles as holding tanks.

Most probable number (MPN) method was used for cell enumerations using the modified Postgate’s B (MPB) medium, standard bacterial nutrient broth, and phenol red dextrose medium for SRB, general heterotrophic bacteria (GHB) and APB, respectively. The MPN procedure can be found elsewhere (Wang et al. 2020b). Biotechnology Solutions (Houston, TX, USA) was the vendor of all these MPN liquid culture media.

After incubation, a confocal laser scanning microscope (CLSM) machine (Model LSM 510, Carl Zeiss, Jena, Germany) was employed to detect live and dead sessile cells on the coupons (Wang et al. 2022b). According to the ASTM G1–03 standard (ASTM, 2003), a fresh Clarke’s solution was used then to clean the coupon surfaces completely before weighing. The coupons were than scanned under an InfiniteFocus microscope (IFM) machine (Model ALC13, Alicona Imaging GmbH, Graz, Austria) to locate MIC pits. The images of surface morphologies before and after coupon surface cleaning were examined under a scanning electron microscope (SEM) machine (Model S-2460 N, Hitachi, Tokyo, Japan).

## Results and discussion

**Fig. 3:**
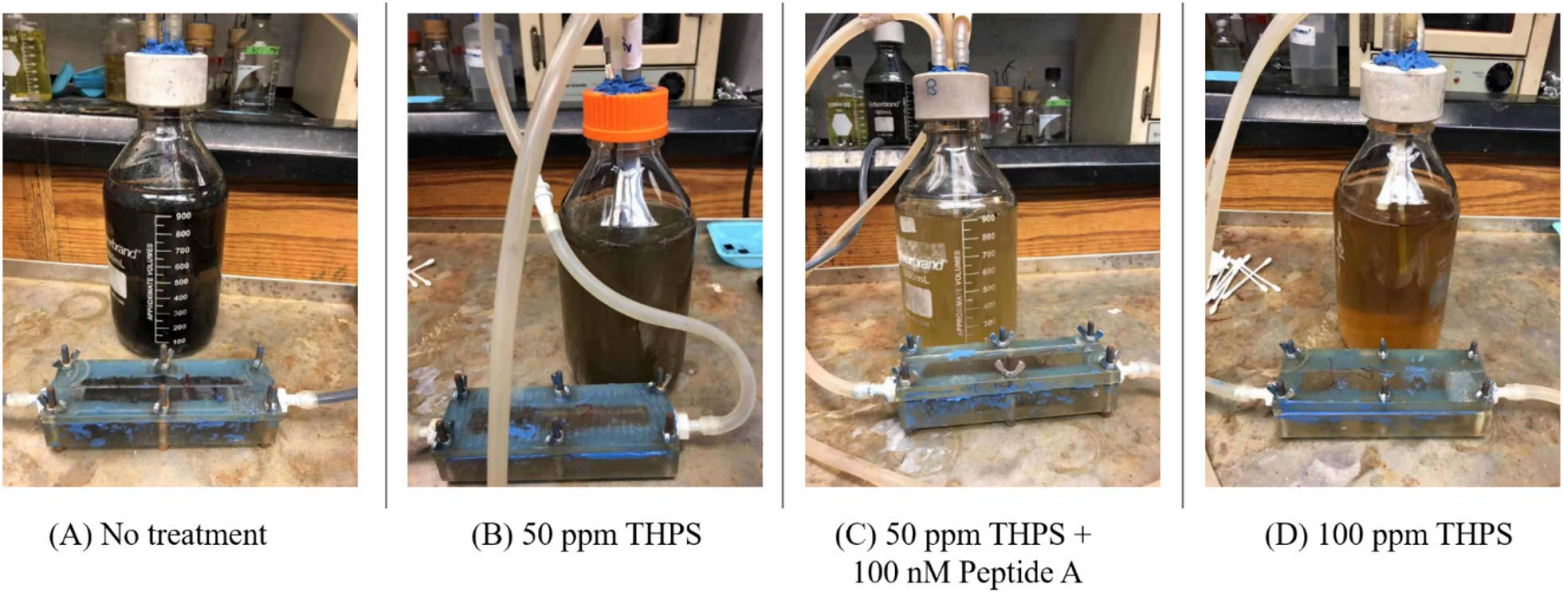
Broth colors in 1 L anaerobic bottles used as holding tanks in flow loops after 7-day incubation with different biocide treatments

**Fig. 4:**
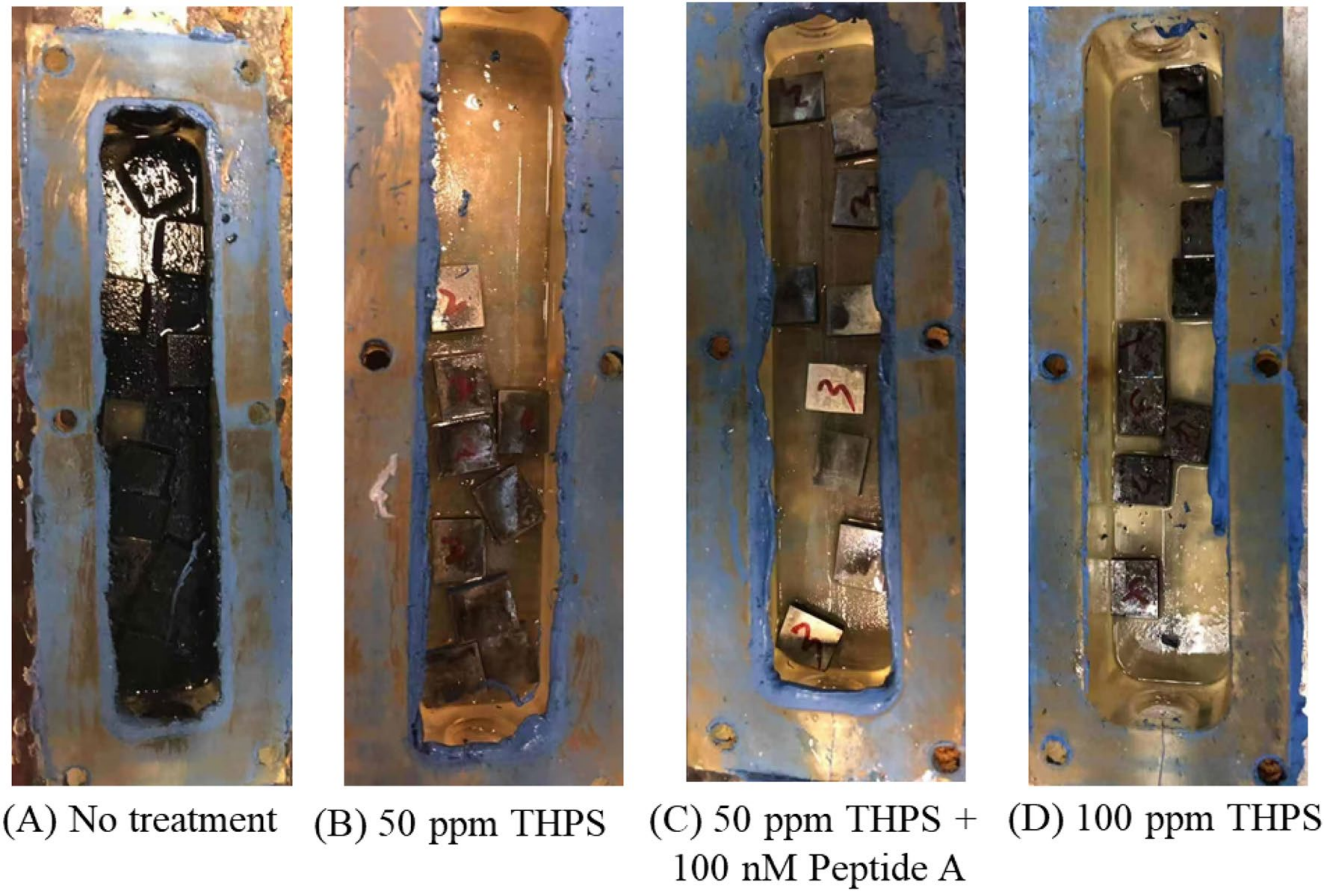
Coupon surfaces after 7-day incubation with different biocide treatments

Figures 3 and 4 show the culture broth colors and coupon surfaces after the 7-day incubation with and without biocide treatment. Judging from the iron sulfide black color, with the treatment of 50 ppm or 100 ppm THPS, or the combination treatment of 50 ppm THPS + 100 nM Peptide A, the broth colors and coupon surfaces appeared less dark than without treatment (Fig. 3), indicating growth inhibition due to biocide treatment.

**Fig. 5:**
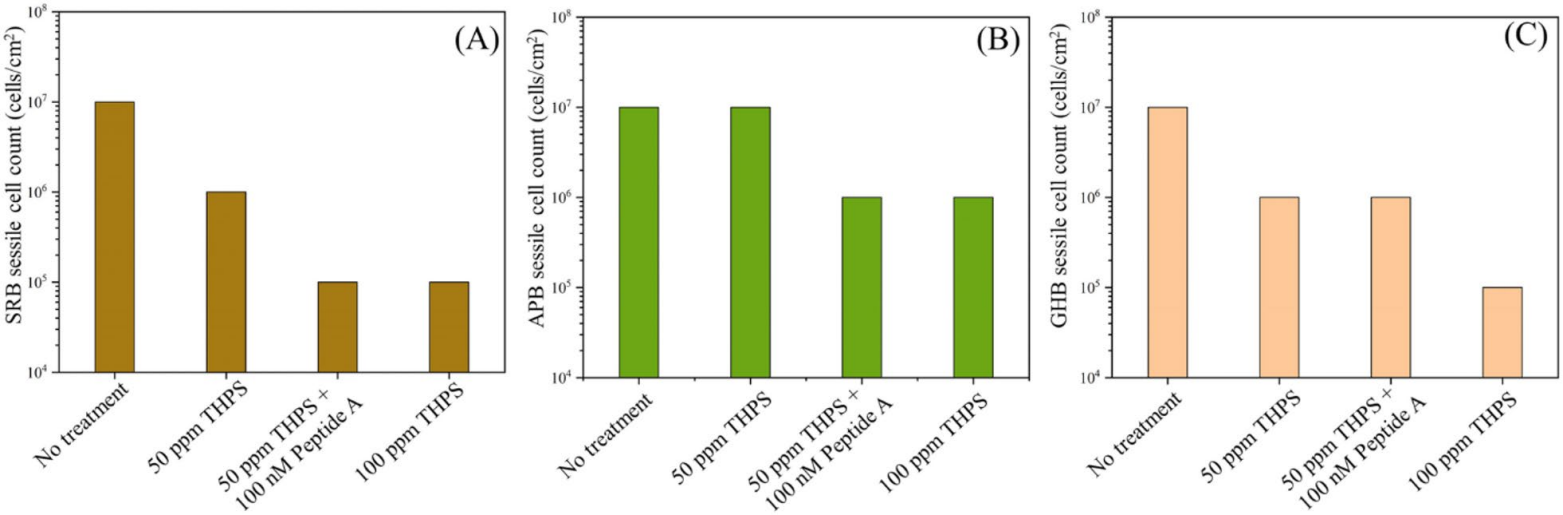
Sessile cell counts of **A** SRB, **B** APB and **C** GHB in flow loop bioreactors after 7-day biofilm prevention test with different biocide treatments

Figure 5 shows sessile cell counts. The no treatment coupon (control) surface had the same sessile cell count of 10^7^ cells/cm^2^ for SRB, APB and GHB. With 50 ppm THPS in the EASW, 1-log reduction in SRB and 1-log reduction in GHB sessile cell counts were achieved, but there was no reduction in APB. With 50 ppm THPS + 100 nM Peptide A combination in EASW, an additional 1-log reduction in SRB and additional 1-log reduction in APB sessile cell counts were obtained, compared to 50 ppm THPS alone treatment.

**Fig. 6:**
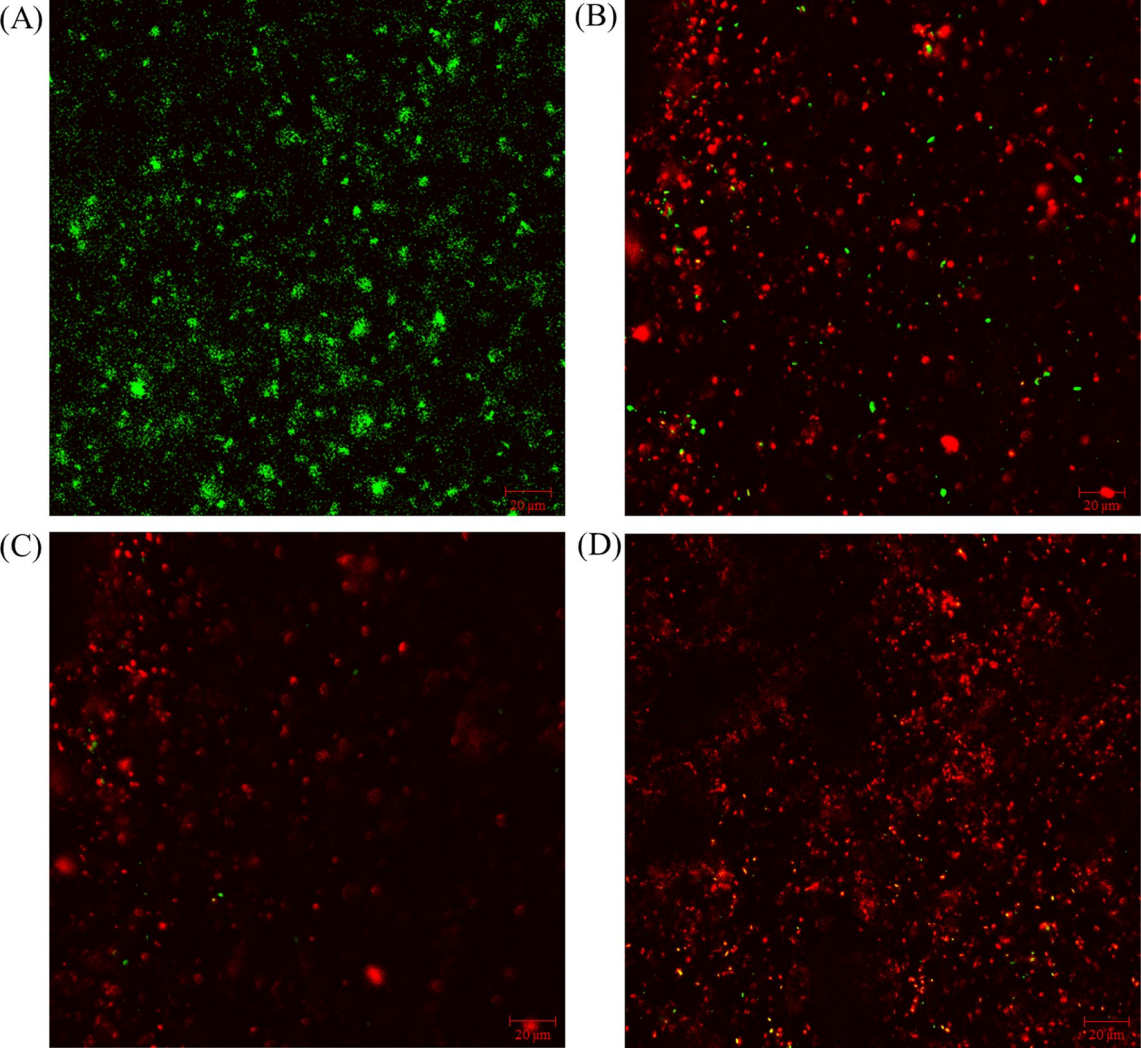
CLSM images of biofilm on C1018 carbon steel surface after 7-day incubation in flow loop bioreactors: **A** no treatment, **B** 50 ppm THPS, **C** 50 ppm THPS + 100 nM Peptide A, **D** 100 ppm THPS

Figure 6 shows the CLSM images of the Consortium IIe biofilms on the C1018 coupons after the 7-day biofilm prevention test. Figure 6A shows that live cells (green) were numerous without biocide treatment (control). When the 50 ppm THPS was used, plenty of dead sessile cells (red) are seen, but some live cells still can be seen in Fig. 6B. With 50 ppm THPS + 100 nM Peptide A in EASW, there was less biomass on the coupon and fewer live cells, exhibiting better efficacy than 50 ppm THPS alone treatment. This outcome was similar to that from 100 ppm THPS alone treatment, but there was a noticeable difference: there were fewer attached dead cells when 100 nM Peptide A was used as a biocide enhancer. This suggests that non-biocidal Peptide A had a biofilm dispersal effect (Jia et al. 2019b).

**Fig. 7:**
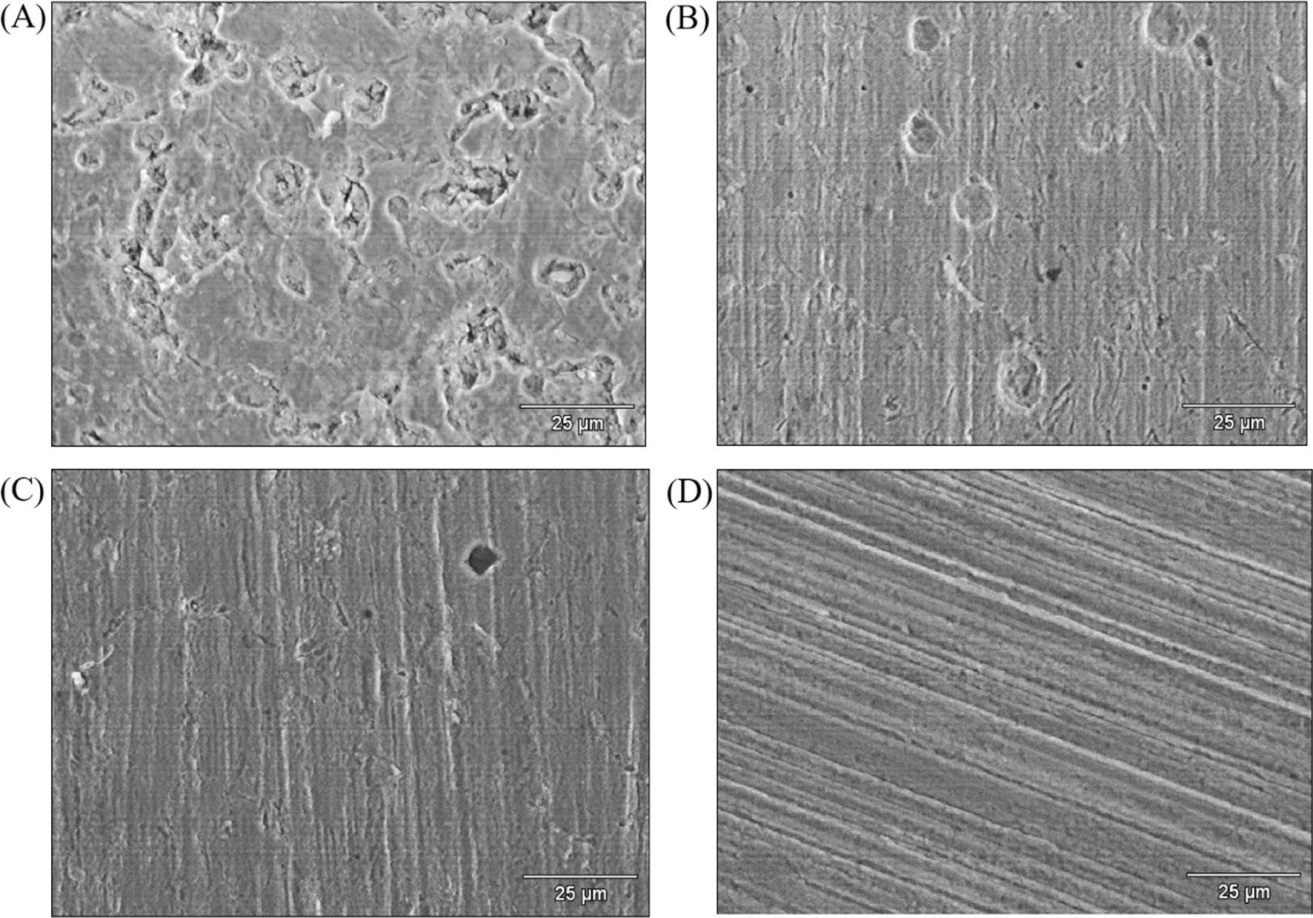
SEM images of corrosion pits on C1018 carbon steel coupons after 7-day incubation in flow loop bioreactors: **A** no treatment, **B** 50 ppm THPS, **C** 50 ppm THPS + 100 nM Peptide A, **D** 100 ppm THPS

Corrosion pit images are shown in Fig. 7 for different biocide treatments. Figure 7A indicates that MIC pits are visible on the no treatment coupon (control), which lost parallel polishing lines due to corrosion damage. With 50 ppm THPS alone in EASW (Fig. 7B), fewer corrosion pits can be found on the coupon surfaces and polishing lines are clearly visible. The 50 ppm THPS + 100 nM Peptide A combination treatment (Fig. 7C) led to fewer and smaller corrosion pits on coupon surfaces. The pristine polishing lines are seen for the coupon with 100 ppm THPS treatment in Fig. 7D. The results of SEM pit images were consistent with the CLSM images and sessile cell count results, suggesting fewer sessile cells leading to less MIC pitting.

**Fig. 8:**
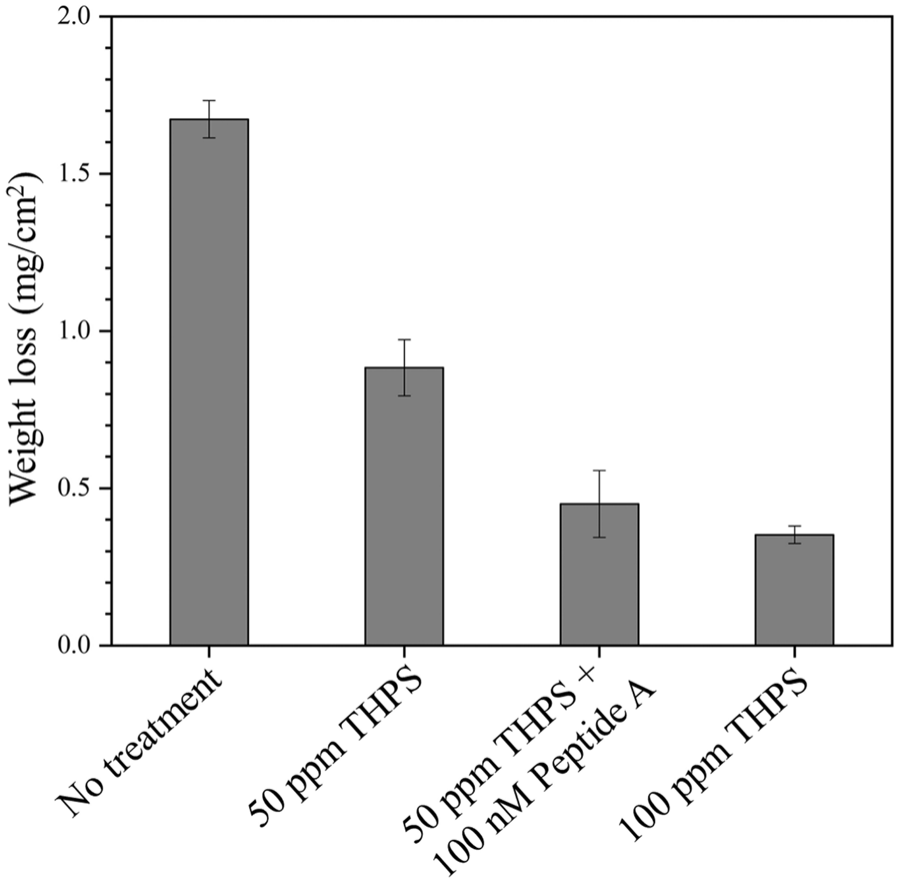
Weight losses of C1018 carbon steel coupons with different biocide treatments after 7-day incubation in flow loop bioreactors (error bars represent standard deviation from three independent samples)

Figure 8 summarizes MIC weight loss data. Each weight loss data point represents the average from six replicate coupons from two replicate experimental runs. The weight loss was 1.7 mg/cm^2^ (equivalent 0.11 mm/*y* uniform corrosion rate) without treatment. With 50 ppm THPS alone treatment, it reduced to 0.9 mg/cm^2^ (0.060 mm/*y*). The 50 ppm THPS + 100 nM Peptide A combination treatment reduced the weight loss further to 0.5 mg/cm^2^ (0.033 mm/*y*), which was only slightly larger than the 0.4 mg/cm^2^ (0.027 mm/*y*) weight loss for 100 ppm THPS alone treatment. This means 100 nM Peptide A effectively reduced the THPS dosage from 100 to 50 ppm in terms of weight loss reduction. The 100 nM Peptide A enhancement decreased weight loss by 71% and 44% compared to no treatment (control) and 50 ppm THPS alone treatment outcomes, respectively.

**Fig. 9:**
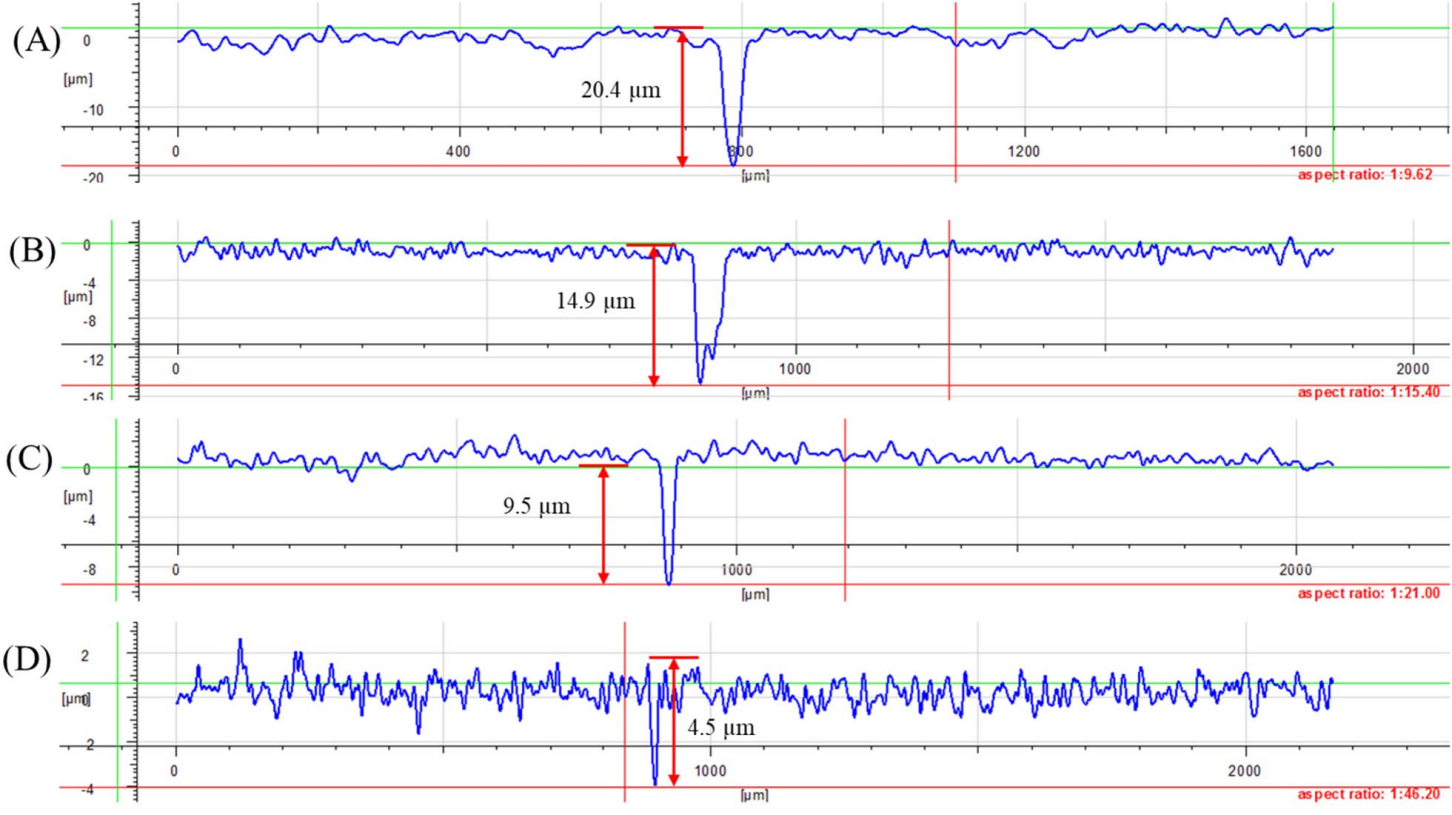
Pit depths of C1018 carbon steel coupons after 7-day biofilm prevention test in flow loop bioreactors: **A** no treatment, **B** 50 ppm THPS, **C** 50 ppm THPS + 100 nM Peptide A, **D** 100 ppm THPS

Figure 9 shows the pit depths with different biocide treatments after the 7-day incubation in the flow loop bioreactors. The (maximum) pit depth for the no treatment control coupon was 20.4 µm. With 50 ppm THPS, it was lowered to 14.9 µm. After 100 nM Peptide A enhancement, the 50 ppm THPS + 100 nM Peptide A combination achieved additional 36% reduction, which lowered the pit depth further to 9.5 µm. The pit depth decreased to 4.5 µm when 100 ppm THPS was in EASW, better than the outcome with 50 ppm THPS + 100 nM Peptide A combination treatment. The mechanism of how Peptide A works as biofilm dispersal agent is still unknown, it very likely works as a signaling agent due to its ultra-low dosage (Jia et al. 2019b). Like d-amino acids, for a recalcitrant biofilm on carbon steel like the one in this work, Peptide A requires a biocidal stress to exhibit a biofilm dispersal effect (Li et al. 2016; Jia et al. 2019b).

## Conclusions

In this work, 50 ppm THPS enhanced by 100 nM Peptide A had considerably better efficacy than 50 ppm THPS alone treatment in the mixed-culture biofilm prevention in the flow system. Additional 44% reduction in weight loss and 36% abatement in pit depth were achieved with 50 ppm THPS treatment enhanced by 100 nM Peptide A. The SEM and CLSM images and sessile cell count were consistent with pit depth and weight loss data trends. The non-biocidal biocide enhancer Peptide A in the presence of a biocidal stress exhibited biofilm dispersal effect. Peptide A at a sub-ppm concentration is a promising biocide enhancer in both flow and static MIC systems.

## Data Availability

Not applicable.

## References

[CR1] Ahmadi A, Schaffie M, Manafi Z, Ranjbar M (2010). Electrochemical bioleaching of high grade chalcopyrite flotation concentrates in a stirred bioreactor. Hydrometallurgy.

[CR2] ASTM G1–03 (2003) Standard Practice for Preparing, Cleaning and Evaluating Corrosion Test Specimens. ASTM

[CR3] Barapatre A, Aadil KR, Jha H (2016). Synergistic antibacterial and antibiofilm activity of silver nanoparticles biosynthesized by lignin-degrading fungus. Bioresour Bioprocess.

[CR4] Bimestre TA, Júnior JAM, Canettieri EV, Tuna CE (2022). Hydrodynamic cavitation for lignocellulosic biomass pretreatment: a review of recent developments and future perspectives. Bioresour Bioprocess.

[CR5] Chang Y-J, Chang Y-T, Hung C-H (2014). Microbial community analysis of anaerobic bio-corrosion in different ORP profiles. Int Biodeterior Biodegrad.

[CR6] Di Somma A, Moretta A, Canè C (2020). Antimicrobial and antibiofilm peptides. Biomolecules.

[CR7] Dong Y, Jiang B, Xu D (2018). Severe microbiologically influenced corrosion of S32654 super austenitic stainless steel by acid producing bacterium *Acidithiobacillus caldus* SM-1. Bioelectrochemistry.

[CR8] Donlan RM (2002). Biofilms: microbial life on surfaces. Emerg Infect Dis.

[CR9] Gieg LM, Jack TR, Foght JM (2011). Biological souring and mitigation in oil reservoirs. Appl Microbiol Biotechnol.

[CR10] Gu T, Wang D, Lekbach Y, Xu D (2021). Extracellular electron transfer in microbial biocorrosion. Curr Opin Electrochem.

[CR11] Hussein HA, Syamsumir DF, Radzi SAM (2020). Phytochemical screening, metabolite profiling and enhanced antimicrobial activities of microalgal crude extracts in co-application with silver nanoparticle. Bioresour Bioprocess.

[CR12] Jia R, Wang D, Jin P (2019). Effects of ferrous ion concentration on microbiologically influenced corrosion of carbon steel by sulfate reducing bacterium *Desulfovibrio vulgaris*. Corros Sci.

[CR13] Jia R, Yang D, Dou W (2019). A sea anemone-inspired small synthetic peptide at sub-ppm concentrations enhanced biofilm mitigation. Int Biodeterior Biodegrad.

[CR14] Kabir H, Munir K, Wen C, Li Y (2021). Recent research and progress of biodegradable zinc alloys and composites for biomedical applications: biomechanical and biocorrosion perspectives. Bioact Mater.

[CR15] Kahrilas GA, Blotevogel J, Stewart PS, Borch T (2015). Biocides in hydraulic fracturing fluids: a critical review of their usage, mobility, degradation, and toxicity. Environ Sci Technol.

[CR16] Kaliyaraj D, Rajendran M, Angamuthu V (2019). Bioleaching of heavy metals from printed circuit board (PCB) by *Streptomyces albidoflavus* TN10 isolated from insect nest. Bioresour Bioprocess.

[CR17] Kijkla P, Wang D, Mohamed ME (2021). Efficacy of glutaraldehyde enhancement by d-limonene in the mitigation of biocorrosion of carbon steel by an oilfield biofilm consortium. World J Microbiol Biotechnol.

[CR18] Kip N, van Veen JA (2015). The dual role of microbes in corrosion. ISME J.

[CR19] Kolodkin-Gal I, Romero D, Cao S (2010). d-amino acids trigger biofilm disassembly. Science.

[CR20] Kumar AK, Sharma S (2017). Recent updates on different methods of pretreatment of lignocellulosic feedstocks: a review. Bioresour Bioprocess.

[CR21] Lekbach Y, Dong Y, Li Z (2019). Catechin hydrate as an eco-friendly biocorrosion inhibitor for 304L stainless steel with dual-action antibacterial properties against *Pseudomonas aeruginosa* biofilm. Corros Sci.

[CR22] Li Y, Jia R, Al-Mahamedh HH (2016). Enhanced biocide mitigation of field biofilm consortia by a mixture of d-amino acids. Front Microbiol.

[CR23] Li S, Show PL, Ngo HH, Ho S-H (2022). Algae-mediated antibiotic wastewater treatment: a critical review. Environ Sci Ecotechnology.

[CR24] Liduino VS, Cravo-Laureau C, Noel C (2019). Comparison of flow regimes on biocorrosion of steel pipe weldments: community composition and diversity of biofilms. Int Biodeterior Biodegrad.

[CR25] Liu T, Cheng YF, Sharma M, Voordouw G (2017). Effect of fluid flow on biofilm formation and microbiologically influenced corrosion of pipelines in oilfield produced water. J Pet Sci Eng.

[CR26] Moradi M, Ye S, Song Z (2019). Dual role of *Psudoalteromonas piscicida* biofilm for the corrosion and inhibition of carbon steel in artificial seawater. Corros Sci.

[CR27] Park HS, Chatterjee I, Dong X (2011). Effect of sodium bisulfite injection on the microbial community composition in a brackish-water-transporting pipeline. Appl Environ Microbiol.

[CR28] Parthipan P, Elumalai P, Ting YP (2018). Characterization of hydrocarbon degrading bacteria isolated from Indian crude oil reservoir and their influence on biocorrosion of carbon steel API 5LX. Int Biodeterior Biodegrad.

[CR29] Qian H, Zhang D, Lou Y (2018). Laboratory investigation of microbiologically influenced corrosion of Q235 carbon steel by halophilic archaea *Natronorubrum tibetense*. Corros Sci.

[CR30] Rasheed PA, Jabbar KA, Rasool K (2019). Controlling the biocorrosion of sulfate-reducing bacteria (SRB) on carbon steel using ZnO/chitosan nanocomposite as an eco-friendly biocide. Corros Sci.

[CR31] Sharma M, Liu H, Chen S (2018). Effect of selected biocides on microbiologically influenced corrosion caused by *Desulfovibrio ferrophilus* IS5. Sci Rep.

[CR32] Sherar BWA, Power IM, Keech PG (2011). Characterizing the effect of carbon steel exposure in sulfide containing solutions to microbially induced corrosion. Corros Sci.

[CR33] Song X, Yang Y, Yu D (2016). Studies on the impact of fluid flow on the microbial corrosion behavior of product oil pipelines. J Pet Sci Eng.

[CR34] Stoodley P, Cargo R, Rupp CJ (2002). Biofilm material properties as related to shear-induced deformation and detachment phenomena. J Ind Microbiol Biotechnol.

[CR35] Tan Z, Shi Y, Xing B (2019). The antimicrobial effects and mechanism of ε-poly-lysine against *Staphylococcus aureus*. Bioresour Bioprocess.

[CR36] Tang H-Y, Yang C, Ueki T (2021). Stainless steel corrosion via direct iron-to-microbe electron transfer by *Geobacter* species. ISME J.

[CR37] Tribedi P, Gupta AD, Sil AK (2015). Adaptation of Pseudomonas sp. AKS2 in biofilm on low-density polyethylene surface: an effective strategy for efficient survival and polymer degradation. Bioresour Bioprocess..

[CR38] Unsal T, Wang D, Kumseranee S (2021). Assessment of 2, 2-dibromo-3-nitrilopropionamide biocide enhanced by d-tyrosine against zinc corrosion by a sulfate reducing bacterium. Ind Eng Chem Res.

[CR39] Vigneron A, Alsop EB, Chambers B (2016). Complementary microorganisms in highly corrosive biofilms from an offshore oil production facility. Appl Env Microbiol.

[CR40] Wang J, Li C, Zhang X (2019). Corrosion behavior of *Aspergillus niger* on 7075 aluminum alloy and the inhibition effect of zinc pyrithione biocide. J Electrochem Soc.

[CR41] Wang D, Liu J, Jia R (2020). Distinguishing two different microbiologically influenced corrosion (MIC) mechanisms using an electron mediator and hydrogen evolution detection. Corros Sci.

[CR42] Wang D, Ramadan M, Kumseranee S (2020). Mitigating microbiologically influenced corrosion of an oilfield biofilm consortium on carbon steel in enriched hydrotest fluid using 2,2-dibromo-3-nitrilopropionamide (DBNPA) enhanced by a 14-mer peptide. J Mater Sci Technol.

[CR43] Wang D, Ivanova SA, Hahn R, Gu T (2022). Evaluation of trehalase as an enhancer for a green biocide in the mitigation of *Desulfovibrio vulgaris* biocorrosion of carbon steel. Bioprocess Biosyst Eng.

[CR44] Wang J, He W, Tan W-S, Cai H (2022). The chitosan/carboxymethyl cellulose/montmorillonite scaffolds incorporated with epigallocatechin-3-gallate-loaded chitosan microspheres for promoting osteogenesis of human umbilical cord-derived mesenchymal stem cell. Bioresour Bioprocess.

[CR45] Wei B, Xu J, Fu Q (2021). Effect of sulfate-reducing bacteria on corrosion of X80 pipeline steel under disbonded coating in a red soil solution. J Mater Sci Technol.

[CR46] Wu Y, Guo H, Rahman MdS (2021). Biological pretreatment of corn stover for enhancing enzymatic hydrolysis using *Bacillus* sp. P3. Bioresour Bioprocess.

[CR47] Yang C, Dou W, Pittman CC (2020). Microbiologically influenced corrosion behavior of friction stir welded S32654 super austenitic stainless steel in the presence of *Acidithiobacillus caldus* SM-1 biofilm. Mater Today Commun.

[CR48] Zlotkin A (2016) Dispersion and detachment of cell aggregates. US Patent No. 9284351 B2.

